# Genetic Variation in Intercellular Adhesion Molecule-1 (*ICAM-1*): candidate gene in susceptibility to malaria in the Indian population

**DOI:** 10.1186/1755-8166-7-S1-P106

**Published:** 2014-01-21

**Authors:** Anuroopa Gupta, Harish Padh

**Affiliations:** 1 Department of Cell and Molecular Biology, B.V. Patel Pharmaceutical Education and Research Development (PERD) Centre, Ahmedabad-380 054, Gujarat, India

## Background

Plasmodium falciparum malaria is the most severe form of malaria causing morbidity and mortality worldwide. The key event in P. falciparum pathogenesis is the sequestration of the infected erythrocytes to the capillary endothelium, mediated by the receptor, Plasmodium falciparum erythrocyte membrane protein-1 (PfEMP1). PfEMP1 has distinct characteristics of binding to varied host ligands including Inter Cellular Adhesion Molecule (*ICAM-1*). Polymorphisms in the gene encoding *ICAM-1* influence the rate of progression or confer protection from or susceptibility to malaria parasite infection. The objective of our study was to evaluate the frequency of *ICAM-1* SNP in exon 6 (rs5498, K469E) in the Indian population and compare it with the global population influenced by the parasitic infection.

## Materials and methods

The genotyping for *ICAM-1* SNP rs5498 was performed by Restriction Fragment Length Polymorphism (RFLP) PCR. Analysis of *ICAM-1* variants was performed in 200 healthy subjects.

## Results

The allelic frequency for the variant ICAM1-Aallele was 59% in healthy control subjects. Chi square analysis reveals that it does not follow the Hardy Weinberg Equilibrium. Comparison with literature data indicates that frequency distribution in Indian population is similar to German, Swedish and Danish populations while it is significantly different from Finnish and Japanese populations. The genotypic frequency of AA was 35%, AG was 49% and GG was 16% in healthy control subjects.

## Conclusions

The frequency data from the above study will help in understanding the role of genetic variations in *ICAM-1* adhesion molecule in the falciparum malaria pathogenesis in the Indian population. Upon comparison, a noteworthy difference in the genotype frequency of Nigerian children (p<0.001) was evinced. This data can be useful for predicting the potential risk of malaria in world population.

